# Placental Histopathology and Clinical Presentation of Severe Congenital Zika Syndrome in a Human Immunodeficiency Virus-Exposed Uninfected Infant

**DOI:** 10.3389/fimmu.2017.01704

**Published:** 2017-12-07

**Authors:** Kíssila Rabelo, Regina Célia de Souza Campos Fernandes, Luiz José de Souza, Thais Louvain de Souza, Flávia Barreto dos Santos, Priscila Conrado Guerra Nunes, Elzinandes Leal de Azeredo, Natália Gedeão Salomão, Gisela Freitas Trindade, Carlos A. Basílio-de-Oliveira, Jorge José de Carvalho, Enrique Medina-Acosta, Marciano Viana Paes

**Affiliations:** ^1^Laboratório de Ultraestrutura e Biologia Tecidual, Universidade do Estado do Rio de Janeiro, Rio de Janeiro, Brazil; ^2^Faculdade de Medicina de Campos, Campos dos Goytacazes, Brazil; ^3^Laboratório de Biotecnologia, Universidade Estadual do Norte Fluminense, Campos dos Goytacazes, Brazil; ^4^Laboratório de Imunologia Viral, Instituto Oswaldo Cruz, Rio de Janeiro, Brazil; ^5^Laboratório Interdisciplinar de Pesquisas Médicas, Instituto Oswaldo Cruz, Rio de Janeiro, Brazil; ^6^Laboratório de Tecnologia Virológica, Biomanguinhos, Rio de Janeiro, Brazil; ^7^Anatomia Patológica, Universidade Federal do Estado do Rio de Janeiro, Rio de Janeiro, Brazil

**Keywords:** Zika virus, placenta, congenital Zika syndrome, histopathology, microcephaly, human immunodeficiency virus

## Abstract

In the large Zika virus (ZIKV) epidemic that occurred in Brazil in 2015, the intrauterine fetal exposure to ZIKV was associated with a significant risk of developing microcephaly and neurological disorders in the infected infants. ZIKV-associated disease has since been reported in 24 countries in the Americas. At present, definitive evidence is lacking regarding the intrauterine co-exposure to ZIKV and other viral infections and whether the coinfection impacts the risk of acquiring either infection or disease severity. Here, we provide evidence of intrauterine exposure to both ZIKV and human immunodeficiency virus (HIV) infections, causing congenital Zika syndrome in an HIV-exposed uninfected infant. Clinical, imaging and laboratory examinations of the pregnant woman and the newborn were performed. Histopathology, ZIKV/HIV-specific immunoassays, and ultrastructural evaluation of the placenta were performed. The Zika-asymptomatic, HIV-positive pregnant woman underwent ultrasounds revealing fetal cerebral ventriculomegaly, microcephaly, and brain atrophy. Her baby girl was born small for gestational age and with the neurological sequelae of congenital Zika syndrome. The evaluation of the abnormally large term placenta revealed severe damage to the maternal decidua and chorionic villi, cells positive for ZIKV-specific antigens but not for HIV antigens, and intracellular membranous clusters of virus-like particles approximately 25 nm in diameter. The rapid progression and severity of the congenital Zika syndrome may be related to the uncontrolled HIV disease in the mother. The poor inflammatory response observed in the placenta may have reduced the inherent risk of mother-to-child transmission of HIV.

## Introduction

The enveloped, positive-strand RNA Zika flavivirus (ZIKV) can be transmitted to humans through *Aedes* mosquito bites, from mother to child, by unprotected sexual intercourse and by blood transfusion (1). The transplacental transmission of ZIKV is highly neurotropic and teratogenic, resulting in severe congenital microcephaly and a broad spectrum of gross and microscopic neuropathologic abnormalities (2, 3). The estimated absolute risk of a notified microcephaly case varies from 0.03 to 17.1% depending on geographical area, the definition of microcephaly used and the ZIKV infection rate (4).

The ZIKV particle is approximately 25–30 nm and shares many structural similarities with other flaviviruses such as Dengue, West Nile, Japanese encephalitis, and Yellow Fever (5). Its viral genome encodes a polyprotein precursor that is processed into the structural proteins [capsid (C), pre-membrane (prM), and envelope (E)] and seven non-structural proteins (NS1, NS2A, NS2B, NS3, NS4A, NS4B, and NS5) (6). The human immunodeficiency virus (HIV), which belongs to the *Retroviridae* family, features two distinct genotypes, HIV-1 and HIV-2. HIV particles are approximately 120 nm in size and contain two single-stranded RNA copies of 9.2 kb (7).

Within less than 2 years from the emergence of an epidemic of ZIKV in Brazil in 2015, 24 countries in the Americas and the Caribbean have reported cases of microcephaly associated with the mother-to-child intrauterine transmission of ZIKV (8). The cellular and molecular pathways that ZIKV uses to breach the human placental barrier to reach the embryo have not been fully elucidated. ZIKV RNA has been detected in various samples from infected individuals: the amniotic fluid, intervillar space, decidua, and chorionic villi (CV) of the placenta, in addition to fetal tissues (9–12). Other placental cells [syncytiotrophoblasts (STB), cytotrophoblasts (CTB), decidual and endothelial cells, macrophages, and dendritic cells] are also permissive to ZIKV (13, 14). At present, there is no decisive proof of intrauterine co-exposure to ZIKV and other viral or parasitic infections and no data on whether such co-exposure impacts the risk of infection or disease severity. One recent report described a case of ZIKV infection acquired during the first trimester in an HIV-infected pregnant woman from Brazil, which was associated with congenital defects and fetal demise. Unfortunately, evaluation of the placenta was not performed (15).

Here, we provide evidence of the intrauterine exposure to both ZIKV and HIV, infection of the placenta with ZIKV, and the outcome of severe congenital Zika syndrome in an HIV-exposed uninfected infant.

## Case and Methods

### Clinical Presentation

**Figure 1:**
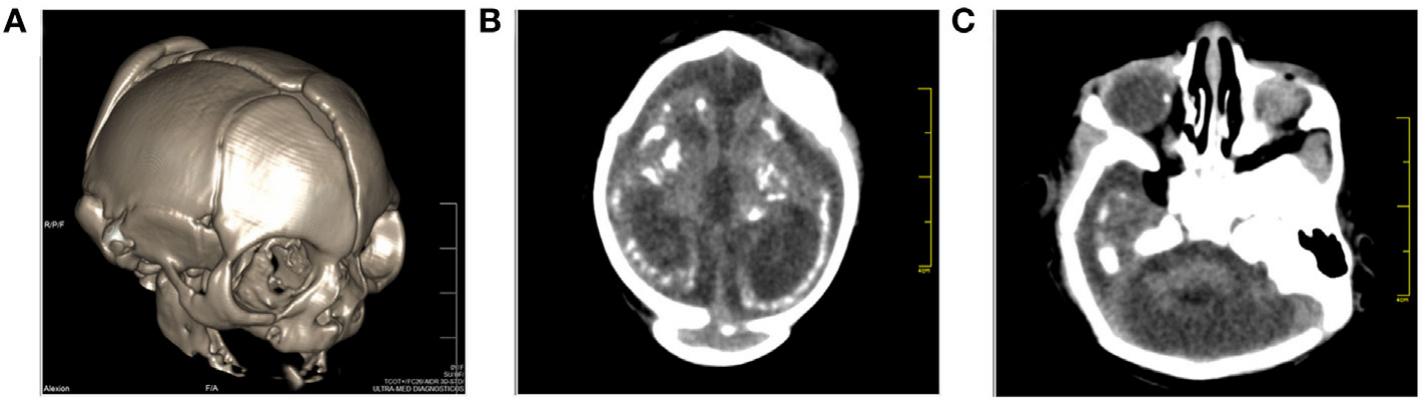
Cranial computed tomography (CT) images of the baby born after Zika virus infection in pregnancy. **(A)** Sagittal localizer CT image of the markedly abnormal skull shape, **(B)** axial CT images showing microcephaly, cerebral atrophy, and multiple dense intracranial periventricular calcifications located in the subcortical white matter at the gray matter–white matter interface, and **(C)** ocular calcification.

A 32-year-old, HIV-positive, primiparous woman from the Northwestern region of the State of Rio de Janeiro, Brazil, had an ultrasound exam at 17 weeks of gestation that was unremarkable about her female fetus. Three weeks later, a second ultrasound revealed fetal cerebral ventriculomegaly. At 24 weeks of gestation, a third ultrasound demonstrated microcephaly and brain atrophy with non-visualization of the cavum septi pellucidi. Prenatal serology was negative for IgM against TORCH antigens (Table S1 in Supplementary Material). The ZIKV infection in the pregnant woman was asymptomatic, but she cited having been bitten by mosquitoes during pregnancy. Two weeks before delivery, the mother had an HIV viral load of 9,323 copies/mL and a CD4+ T-lymphocyte count of 373 cells/μL. Since her HIV diagnosis in the year 2012, the woman had occasionally used antiretroviral treatment consisting of Tenofovir, Lamivudine, and Lopinavir/Ritonavir, but she did not have any AIDS-defining illness. Before delivery, she received Zidovudine for the prevention of mother-to-child transmission of HIV. At 38 weeks of gestation (June 1st, 2016), her baby girl was born by cesarean delivery. She was small for gestational age (below the third percentile: weight 1.375 g, height 35 cm, and cephalic circumference 24.5 cm) and the placenta weighed 325 g (placental coefficient: 0.236; reference value: 0.182 ± 0.023). The newborn physical examination showed a flat midface, low nasal bridge and short nose, overlapping sutures and redundant occipital skin folds, asymmetrical microphthalmia, upper and lower limb contractures, and valgus deformities of the knees. A cranial computed tomography scan revealed cerebral atrophy with the partial collapse of the skull (Figure 1A), ventriculomegaly, supratentorial hydrocephalus, ocular globe asymmetry and multiple intracranial periventricular calcifications (Figure 1B), along with intraocular calcifications (Figure 1C). Indirect ophthalmoscopy revealed atrophy at all quadrants, including the optic nerve in the right eye, and atrophy and peripheral pigmentation in the upper and posterior poles of the retina in the left eye. The echocardiographic evaluation showed a patent foramen ovale. A qPCR test for ZIKV in a liquor sample taken at birth yielded inconclusive results (data not shown). At ages of 2 and 4 months, the infant had an undetectable HIV viral load. When the child was 6 months of age, the mother’s serum tested IgG-positive and IgM-negative for ZIKV and negative for anti-Dengue and anti-Chikungunya viral antibodies (Table S1 in Supplementary Material). The timeline of events and findings is presented in Figure S1 in Supplementary Material.

### Ethical Procedures

All procedures performed during this work were approved by the Ethics Committee of the Oswaldo Cruz Foundation/FIOCRUZ (CAEE: 65924217.4.0000.5248). The mother of the index patient gave written consent and permission for the publication of data and images.

### Sample Collection

At delivery, samples from the index case placenta were collected and fixed following various histopathological techniques. Blood samples from the pregnant woman were collected postpartum. A spinal liquor sample was collected from the infant. Samples were collected at the Hospital Plantadores de Cana, Campos dos Goytacazes, RJ, Brazil. As a reference control, a sample of term placenta from a healthy donor was included.

### Histopathological Analysis

Samples from the placentas were fixed in formalin (10%), dehydrated in ethanol, clarified in xylene and blocked in paraffin resin. Tissue sections were cut (4 µm thick), deparaffinized in three baths of xylene and rehydrated with decreasing concentrations of ethanol (100 to 70%). Sections were stained with hematoxylin and eosin for histological examination and with periodic acid–Schiff to examine extracellular matrix (EM) morphometry. Stained specimens were visualized by light microscopy (Olympus, Tokyo, Japan), and digital images were obtained using Image-Pro Plus software version 4.5. The case and control samples were coded and handled in a blind test.

### Morphometry and Statistical Analysis

The slides were visualized under a light microscope (Olympus), and the EM area was analyzed using Image-Pro Plus software version 4.5. Fifty fields were randomly acquired at 400× magnification from both placentas (Zika-infected and control). The regions stained with PAS were measured, and the percentage of EM was calculated (histological feature/total area of the image). Data were analyzed with GraphPad Prism software v 6.0 (GraphPad Software, CA, USA) using non-parametric statistical tests. Significant differences between the analyzed groups were determined using the Mann–Whitney test with a threshold of *P* < 0.05.

### Immunohistochemistry Procedures

The paraffin-embedded tissues were cut (4 µm), deparaffinized in xylene and rehydrated with alcohol. Antigen retrieval was performed by heating the tissue in the presence of citrate buffer. Next, tissues were blocked for endogenous peroxidase with 3% hydrogen peroxidase in methanol and rinsed in Tris–HCl (pH 7.4). Sections were incubated in Protein Blocker solution (Spring Bioscience, CA, USA) for 5 min at room temperature to reduce non-specific binding. They were then incubated overnight at 4°C with a 1:200 dilution of the mouse monoclonal antibody 4G2 (16), which is specific for the fusion loop at the extremity of domain II of the Flavivirus group antigen envelope (E) protein (16), or the mouse monoclonal IgG antibody against Zika virus non-structural protein NS1 (Arigo Biolaboratories, Taiwan, Republic of China), or with a 1:40 dilution of the mouse monoclonal antibody specific for the p24 protein of HIV (Agilent—Dako, CA, USA). The next day, sections were incubated with a rabbit anti-mouse IgG conjugated to horseradish peroxidase (Spring Bioscience Corporation, CA, USA) for 40 min at room temperature. Reactions were revealed with diaminobenzidine (Agilent) as a chromogen, and the sections were counterstained with Meyer’s hematoxylin (Agilent).

### Immunofluorescence Assay

The paraffin-embedded tissues were processed as above, except for incubation with 1% bovine serum albumin for 30 min, and permeabilized with 0.5% Triton X-100 at room temperature. Slides were co-stained overnight at 4°C with a 1:200 dilution of a mouse monoclonal antihuman proliferating cell nuclear antigen (PCNA) IgG (ThermoFisher, OK, USA) or a mouse monoclonal anti-Zika NS1 IgG, and a rabbit monoclonal antihuman CD11b IgG (Abcam, Cambridge, UK); or with a 1:40 dilution of the mouse monoclonal antibody specific for the p24 protein of HIV (Agilent—Dako, USA). Sections were incubated with Alexa 488-conjugated rabbit anti-mouse IgG, Alexa 555-conjugated goat anti-rabbit IgG, or Alexa 555-conjugated goat anti-mouse IgG (ThermoFisher). Slides were analyzed using a Zeiss LSM 510 Meta confocal microscope (Carl Zeiss, Oberkochen, Germany).

### Electron Microscopy Procedures

Tissue samples were fixed with 2.5% glutaraldehyde in sodium cacodylate buffer (0.1 M, pH 7.2), post-fixed with 1% buffered osmium tetroxide, dehydrated in an acetone series (30, 50, 70, 90, and 100%), and embedded in EPON polymerized at 60°C for 3 days. Ultrathin sections (60–90 nm) were contrasted with uranyl acetate and lead citrate and were visualized using a JEOL 1001 transmission electron microscope (Jeol Ltd., Tokyo, Japan).

## Results

**Figure 2:**
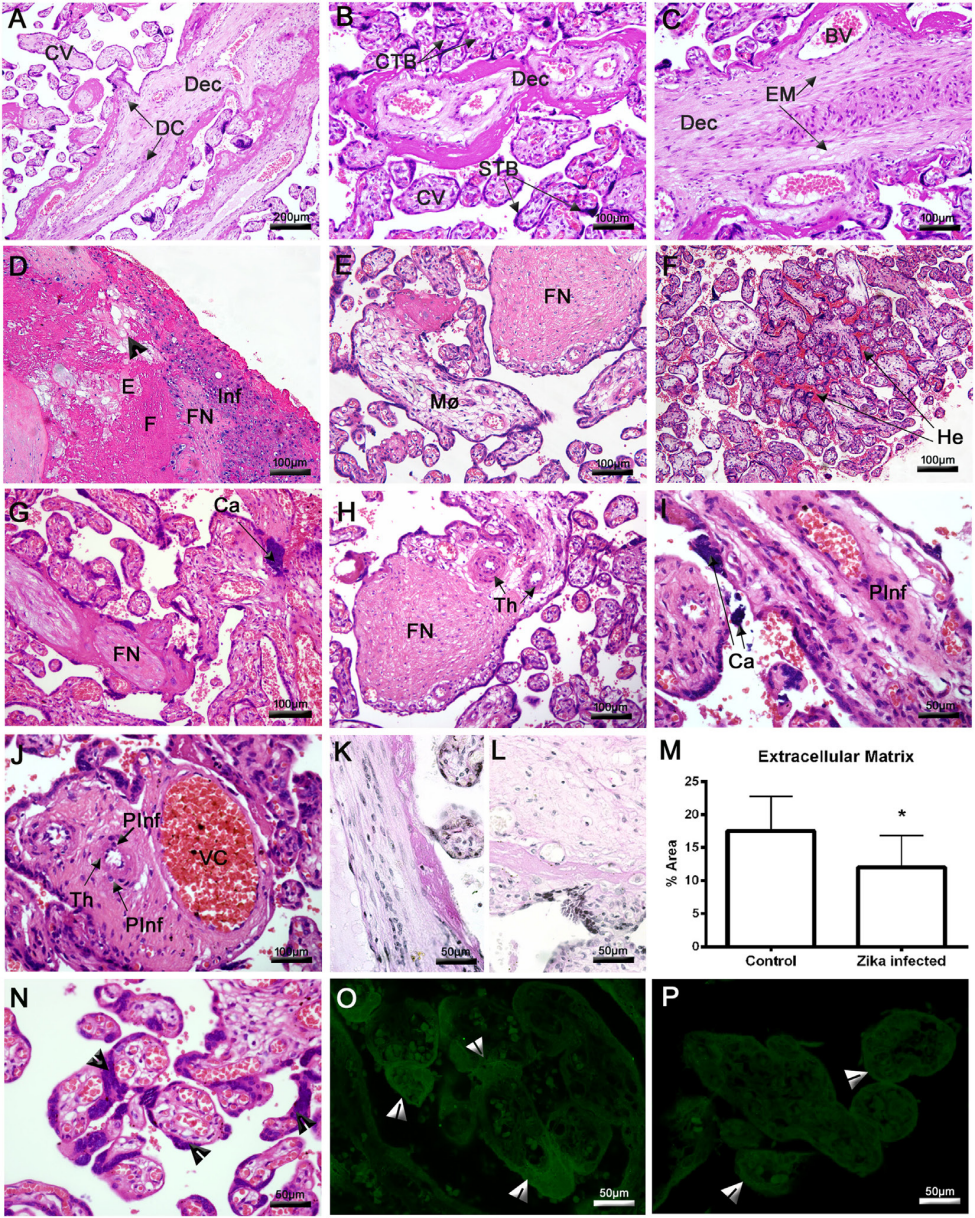
Histopathological analysis of the placenta. **(A–C)** Placenta of a non-ZIKV patient stained with H&E and presenting normal features: maternal decidua (Dec), decidual cells (DC), chorionic villi (CV), syncytiotrophoblasts (STB), cytotrophoblasts (CTB), extracellular matrix (EM), and blood vessels (BV). **(D–J,N)** Sections of ZIKV-infected placental tissue stained with H&E, showing abnormalities in the decidua, including edema **(E)**, fibrosis **(F)**, fibrinoid necrosis (FN), mononuclear inflammatory infiltrate (Inf), macrophages (Mø), endothelial thickening (Th), cellular degeneration [arrowhead in panel **(D)**], calcification (Ca), and other pathological features in CV, such as perivascular inflammatory infiltrate (PInf), vascular congestion (VC), hemorrhage (He), and inordinate proliferative STB [arrowheads in panel **(N)**]. **(K)** Placental sections from a non-ZIKV and **(L)** a ZIKV-infected patient stained with PAS, evidencing highlighting the EM. **(M)** The percent EM area was quantified in both cases; asterisks indicate statistically significant differences between samples: *P < 0.05. **(O)** Immunofluorescence analysis of proliferating cell nuclear antigen expression in the STB of a ZIKV-infected and **(P)** a non-ZIKV patient.

Histopathological analysis of the ZIKV-infected placenta revealed severe damage to the maternal decidua and CV. In the control placenta, we observed a regular structure of decidual parenchyma with typical decidual cells (DC) and capillaries exhibiting regular endothelial cells. Likewise, CV showed normal STB, CTB, and endothelial cells (Figures 2A–C). The most prominent lesion in the placenta from the Zika-infected patient was the presence of large and diffuse areas of fibrinoid necrosis (FN) in the maternal decidua (Figures 2D,E,G,H). The maternal portions of the placenta also presented diffuse edema, fibrosis, vascular endothelial thickening, degeneration, vascular congestion (VC) and focal areas of mononuclear or perivascular inflammatory infiltrates (Figures 2D,E,G–J). The decidua showed dense and heterogeneous calcification, which can be consistent with third-trimester gestation (Figures 2G,I). Since the histopathological analysis indicated degeneration of the decidua, the EM was assessed using PAS staining. Morphological analysis revealed a significant reduction in the total area of EM in the placenta from the Zika-infected patient (12.02% ± 1.33) compared with the control placenta (17.52% ± 1.45) (Figures 2K–M). An investigation of the CV revealed an area of extensive hemorrhage and prominent STB (Figures 2F,N). A higher density of PCNA was observed in the STB of the index placenta compared with the control placenta (Figures 2O,P). PCNA acts as a scaffold to recruit proteins involved in DNA replication or repair.

**Figure 3:**
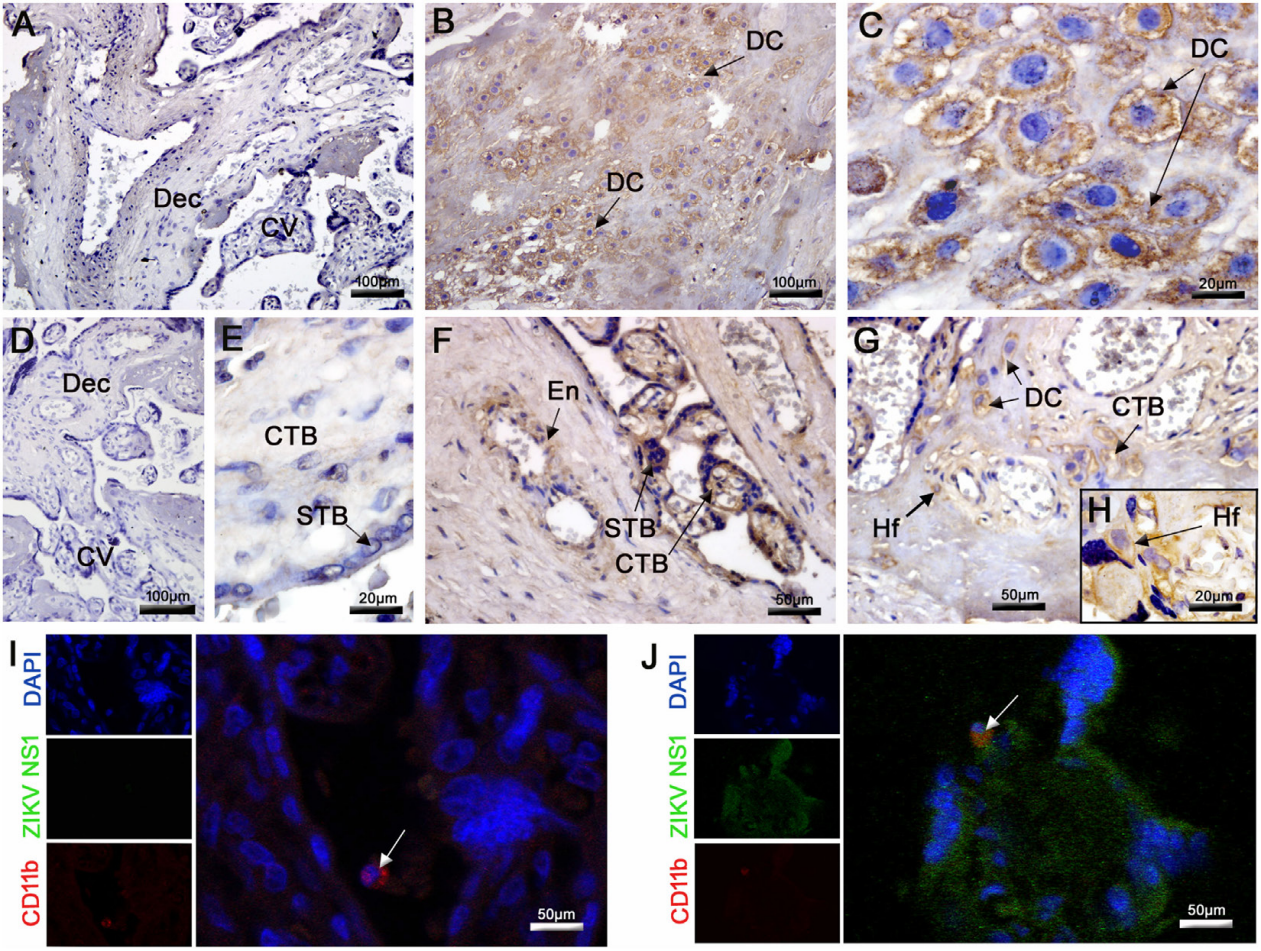
Detection of ZIKV in the placenta. **(A,D,E)** The E and NS1 antigens of ZIKV were not detected by immunohistochemistry in the control placenta. **(B,C)** Detection of ZIKV E protein in decidual cells (DC) by immunohistochemistry in the infected placenta. **(F–H)** The NS1 protein of ZIKV was also detected by immunochemistry in the endothelium (En), DC, syncytiotrophoblasts (STB), cytotrophoblasts (CTB), and Hofbauer cells (Hf). **(I,J)** Co-localization by immunofluorescence of the NS1 protein (fluorescent green) and CD11b for identification of leukocytes (fluorescent red). Nuclei were stained using DAPI (fluorescent blue). **(I)** ZIKV NS1 antigen was not detected in the control placenta. **(J)** Cells presenting dual staining (green and red) were observed in the ZIKV-infected placenta.

Placentas were tested for the presence of ZIKV antigens using an immunohistochemistry assay. Virus antigens were observed only in samples from the ZIKV-infected patient. No immunostaining was observed in samples from the control placenta (Figures 3A,D,E). ZIKV envelope proteins were identified mainly in DC (Figures 3B,C), whereas the NS1 protein was detected in the cytoplasm of several placental cells, such as decidual and endothelial cells in the maternal decidua. In the CV, the NS1 protein was detected in CTB, STB, and Hofbauer cells, which strongly suggests that viral replication occurred in these cells (Figures 3F–H). Further evidence for ZIKV replication in these cells was provided by the positive immunofluorescence signal for NS1 protein (green fluorescence) in several placental cells and its co-localization with CD11b (red fluorescence), used to identify infected mononuclear cells (Figure 3J). As expected, no positive reactions against NS1 were observed in control tissue (Figure 3I). The term placenta was negative for HIV-specific p24 protein as assessed by immunohistochemistry and fluorescence (Figure S2 in Supplementary Material).

**Figure 4:**
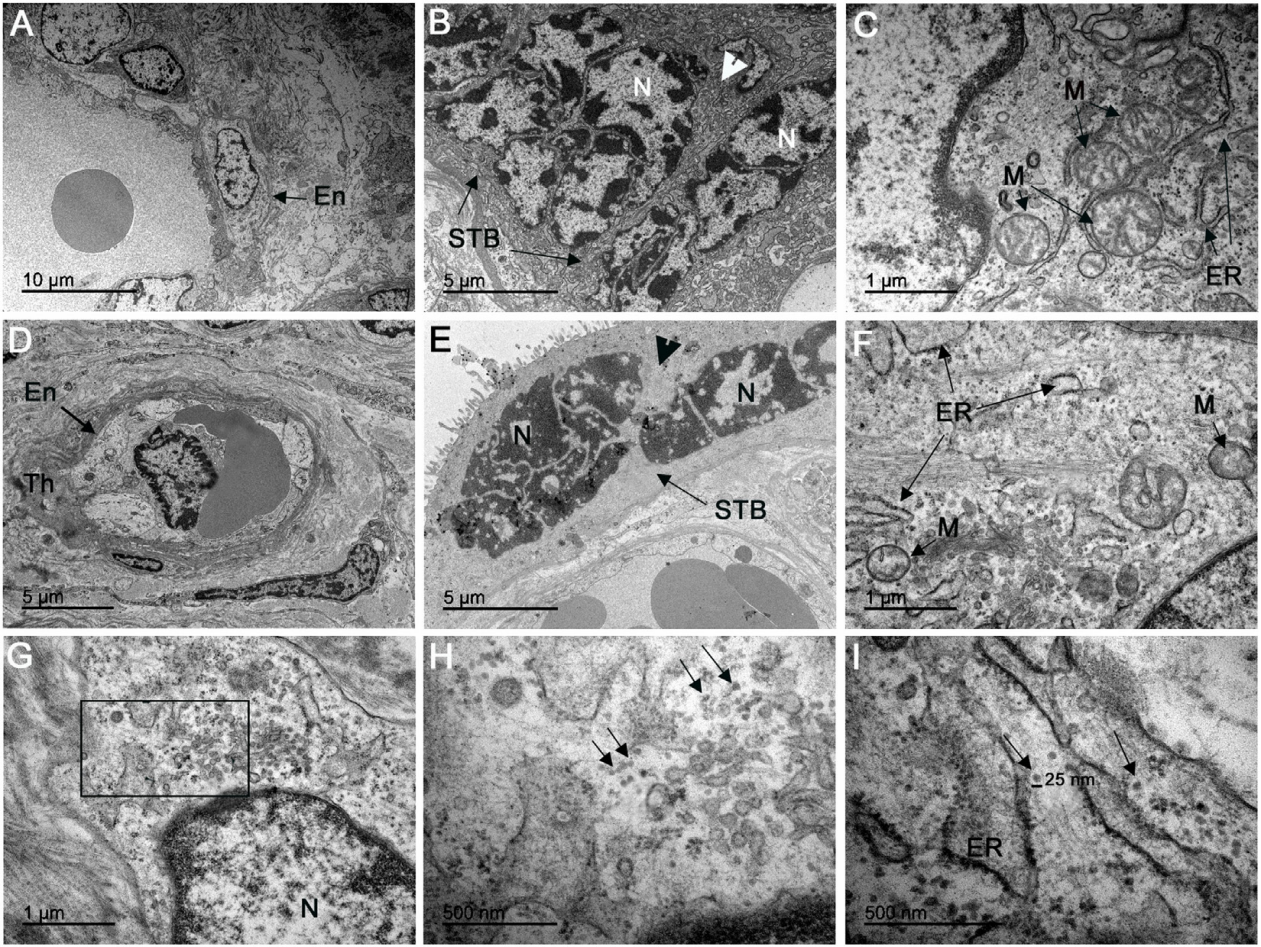
Electron microscopy analysis of ultrathin placental sections showed virus-like particles. **(A–C)** Electron microscopy of ultrathin sections of one non-ZIKV case exhibited regular endothelial cells (En), syncytiotrophoblasts (STB), and CTB organelles, such as mitochondria (M) and endoplasmic reticulum (ER). **(D–F)** Electron micrographs of ZIKV-infected placenta showing thickening of the basement membrane (Th) of the endothelium (En), dispersed chromatin in syncytiotrophoblast nuclei (N) gathered in the vicinity of the nuclear membrane, and rarefied cytoplasm with absent organelles (arrowhead); mitochondria (M) in cytotrophoblasts were smaller, with fewer cristae, and the ER exhibited dilated cisterns. **(G)** Cytotrophoblast of a ZIKV-infected patient with a cluster of virions in the cytoplasm. **(H)** In the same area, at a higher magnitude, we observed these virus-like particles located near disrupted ER. **(I)** Measurement using the scale bar showed that the particles have a diameter of approximately 25 nm, consistent with ZIKV.

No evidence of ultrastructural changes was noted in the control patient (Figures 4A–C). In contrast, the infected placenta showed various alterations such as thickening of the endothelial basement membrane (Figure 4D), the nuclei of the STB with dispersed chromatin aggregated in the vicinity of the nuclear membrane, and rarefied cytoplasm with absent organelles (Figure 4E). Moreover, smaller mitochondria and fewer cristae were observed, and the endoplasmic reticulum (ER) exhibited dilated cisterns in CTB (Figure 4F). Ultrathin sections of placental tissue allowed the identification of clusters with dense virus-like particles, located in damaged cytoplasmic vesicles of CTB (Figure 4G). We also observed these virus-like particles near disrupted areas of the ER. The remains of membranes could be seen at the periphery of these clusters (Figure 4H). These particles were approximately 25 nm in diameter, which is consistent with the physical dimensions of ZIKV (Figure 4I). We did not observe virus-like particles with diameters consistent with mature or immature HIV (range 110–146 nm).

## Discussion

Overall, the clinical and placental evaluations support a diagnosis of intrauterine infection with ZIKV and exposure to HIV infection, with an outcome of severe congenital Zika syndrome in an HIV-exposed uninfected infant. Similar structural abnormalities of the skull and brain have been documented in presumed and confirmed ZIKV infection in pediatric HIV-unexposed cohorts (17, 18). We note that the progression of the fetal disease to microcephaly occurred within a period of 7 weeks in the second trimester, a timeframe that is consistent with the maternal perfusion of the placenta occurring in the late first to the second trimester. We speculate that the ZIKV infection of the fetus occurred early in the second trimester because the extent of the sequelae did not include abnormalities otherwise related to first-trimester development (i.e., holoprosencephaly and craniosynostosis) (18). ZIKV RNA and ZIKV antigens have been detected in several types of placental cells from pregnancies with histopathologic alterations by ZIKV infection and localized ZIKV antigens or RNA in placental all cells (12, 19). Furthermore, published data indicate that infection with another flavivirus, such as Dengue, is associated with a transient decrease in HIV viral load, and no severe Dengue or HIV progression is observed during coinfection (20). This is consistent with our observations of the current HIV-positive pregnant patient infected by ZIKV. Notwithstanding the histopathological damage associated with the ZIKV infection of the placenta and the detectable viral load of HIV in the pregnant woman, the HIV did not infect the placenta. Importantly, a recent study correlating the placental characteristics from pregnancies in HIV-infected-positive and HIV-negative women described no specific lesions in infected placentas (21). Moreover, the birth head circumference of the newborns was similar in the two groups. In addition, vertical transmission was avoided by the use of antiretroviral drugs (21).

Notwithstanding the extent and severity of the gross damage caused to both the placenta and the fetal brain by the ZIKV infection, the severe intrauterine growth deficiency was compatible with life. In cases of maternal coinfection (i.e., Cytomegalovirus and HIV coinfection), in which there is a considerable inflammatory response in the placenta, often one viral infection predisposes the patient to a higher risk of acquiring the other (22). In a recent prospective cohort study in 134 ZIKV-positive/HIV-negative pregnant women with new-onset rash manifestations, neither the severity of maternal ZIKV disease, the virus RNA load nor the prior existence of Dengue antibodies was significantly associated with abnormal birth outcomes (23). We cannot rule out a collateral damage effect from the HIV co-exposure on the placental histopathological abnormalities caused by ZIKV infection.

The findings in the abnormally large term placenta suggest that ZIKV spread from the maternal decidua to the CV and reached the fetus by replication and transmission from cell to cell. This hypothesis is based on the detection of NS1 protein in the cytoplasm of several placental cells, such as decidual and endothelial cells in the maternal decidua, and CTB, STB, and Hofbauer cells, which supports the hypothesis of active viral replication. The occurrence of membranous clusters of viral particles in the cytoplasm of CTB is therefore reminiscent of replication factories. Moreover, the observed enrichment with the PCNA expression signal (a protein involved in DNA replication and repair) (24) in the STB indicates an attempt by the cells to repair the injured DNA. Some studies showed that the binding of PCNA to repair proteins is direct, and that the inhibition of its expression impairs the mechanism of DNA repair (25, 26). Tissue alterations such as FN, edema, calcification, and mononuclear inflammatory infiltrates are commonly seen in ZIKV-infected premature placentas (9, 12, 18). Significantly, diffuse fibrosis, vascular endothelial thickening, cellular degeneration, hemorrhage, and VC had not been previously reported in ZIKV-infected term placentas. Furthermore, we observed infected mononuclear cells that are capable of supporting replicating virus, including dendritic cells and macrophages (Hofbauer cells), which are known to be primary targets of another flavivirus, Dengue (27). However, an *in vitro* study with cells isolated from mature placenta showed that placental macrophages and CTB are permissive to ZIKV infection (14). In addition, Tabata et al. (13) observed that all placental cells, as well as immune cells, in the tissue present receptors capable of binding to the virus and mediating cell entry by endocytosis.

## Concluding Remarks

It is important to note that the viral antigens persisted in the placental tissue months after the infection occurred. Ultimately, the presence of dense, virus-like particles consistent with the size of ZIKV, seen near the ER of CTB, was the most definitive indication of a persistent viral infection in placenta, from the second trimester until delivery. Our results confirm that maternal ZIKV infection during pregnancy can result in placental and fetal injury. Furthermore, in countries where the prevalence of HIV-positive pregnant women is high, such as in Sub-Saharan African nations (5.3% average prevalence), more cases of severe Zika disease in HIV-exposed fetuses are expected to occur if the epidemic potential of ZIKV increases there (28). The present index case serves as evidence for the importance of preparedness.

## Ethics Statement

This study was carried out in accordance with the recommendations of “Ethics Committee of the Oswaldo Cruz Foundation/FIOCRUZ (CAEE: 65924217.4.0000.5248)” with written informed consent from all subjects. All subjects gave written informed consent in accordance with the Declaration of Helsinki. The protocol was approved by the “Ethics Committee of the Oswaldo Cruz Foundation/FIOCRUZ.”

## Author Contributions

KR, MP, RF, and EM-A designed the study. LS, CB-d-O, TS, and RF collected samples and performed clinical and tomography exams. KR performed all research experiments for placental evaluation. FS, PN, EA, NS, and GT optimized or supported immunohistochemical experiments. KR and EM-A wrote the manuscript. KR, MP, CB-d-O, and JC analyzed the experimental results. MP, JC, RF, and TS edited the manuscript. All the authors gave final approval.

## Conflict of Interest Statement

The authors declare that the research was conducted in the absence of any commercial or financial relationships that could be construed as a potential conflict of interest.

## Supplementary Material

Figure S1 Timeline of relevant events, studies and findings in the presented case of intrauterine co-exposure to ZIKV and human immunodeficiency virus (HIV).

Figure S2 Immunohistochemistry and fluorescence for p24 human immunodeficiency virus (HIV) protein. Tissue sections were deparaffinized and rehydrated, and antigen retrieval was performed by heating the tissue in the presence of citrate buffer. Sections were blocked and then incubated overnight at 4°C with a 1:40 dilution of the mouse monoclonal antibody specific for the p24 protein of HIV. The next day, sections were incubated with a rabbit anti-mouse IgG-HRP conjugate for immunohistochemistry or Alexa 555-conjugated goat anti-mouse IgG for immunofluorescence. Reactions were revealed with diaminobenzidine as the chromogen and the sections were counterstained with Meyer’s hematoxylin for immunohistochemistry. **(A,C)** The p24 antigen of HIV was not detected by immunohistochemistry or by immunofluorescence in the control placenta. **(B,D)** The p24 antigen of HIV was not detected by immunohistochemistry or by immunofluorescence in the ZIKV index case placenta.
