# Supplementary figures and images for: Placental Histopathology and Clinical Presentation of Severe Congenital Zika Syndrome in a Human Immunodeficiency Virus-Exposed Uninfected Infant
Source: Front Immunol. 2017 Dec 7;8:1704. doi: 10.3389/fimmu.2017.01704 (PMC5725436; doi:10.3389/fimmu.2017.01704)

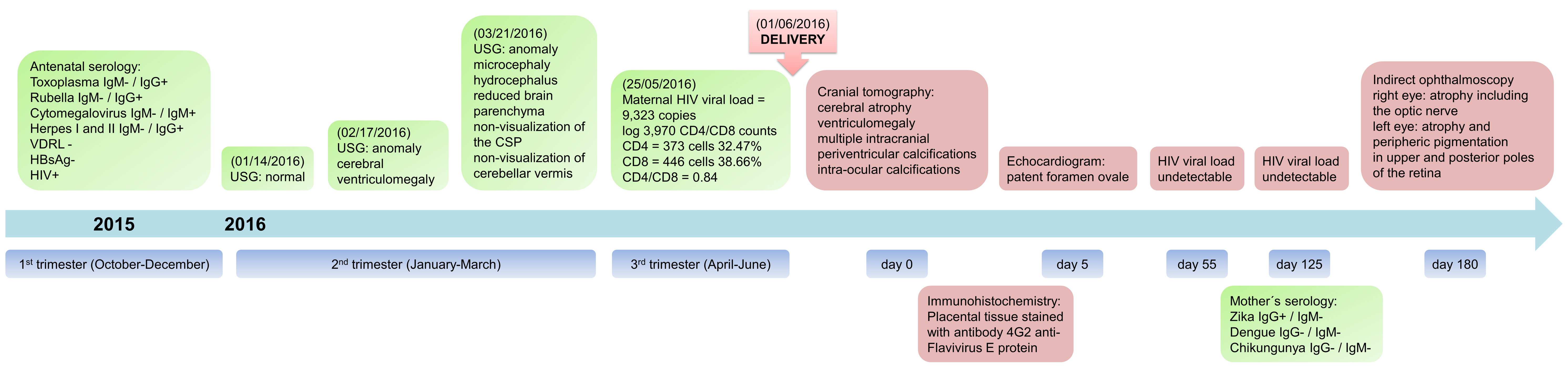

Supplement: Figure S1 — Timeline of relevant events, studies and findings in the presented case of intrauterine co-exposure to ZIKV and human immunodeficiency virus (HIV). [file Image_1.TIF]

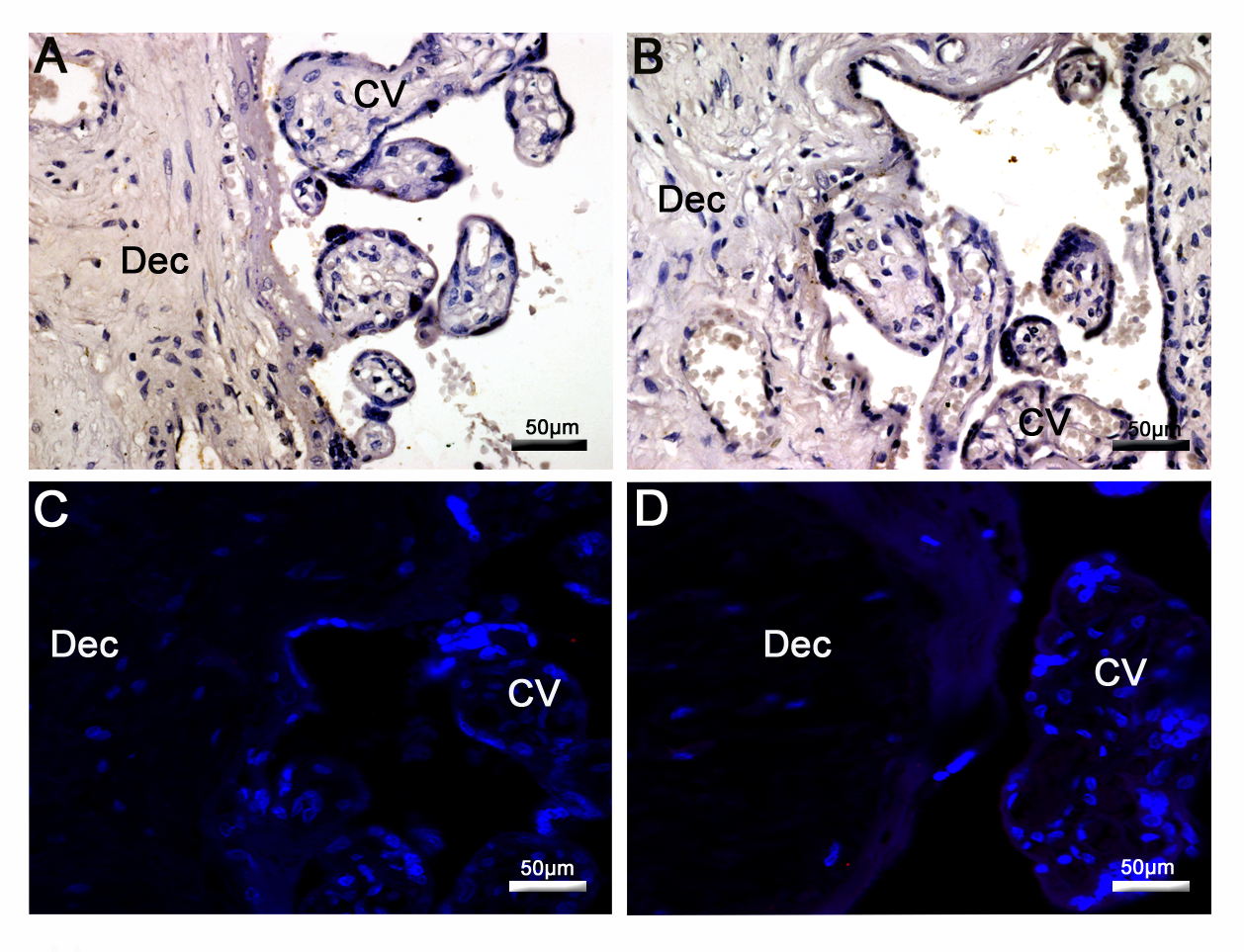

Supplement: Figure S2 — Immunohistochemistry and fluorescence for p24 human immunodeficiency virus (HIV) protein. Tissue sections were deparaffinized and rehydrated, and antigen retrieval was performed by heating the tissue in the presence of citrate buffer. Sections were blocked and then incubated overnight at 4°C with a 1:40 dilution of the mouse monoclonal antibody specific for the p24 protein of HIV. The next day, sections were incubated with a rabbit anti-mouse IgG-HRP conjugate for immunohistochemistry or Alexa 555-conjugated goat anti-mouse IgG for immunofluorescence. Reactions were revealed with diaminobenzidine as the chromogen and the sections were counterstained with Meyer’s hematoxylin for immunohistochemistry. (A,C) The p24 antigen of HIV was not detected by immunohistochemistry or by immunofluorescence in the control placenta. (B,D) The p24 antigen of HIV was not detected by immunohistochemistry or by immunofluorescence in the ZIKV index case placenta. [file Image_2.TIF]
