# Supplementary material for: Placental Histopathology and Clinical Presentation of Severe Congenital Zika Syndrome in a Human Immunodeficiency Virus-Exposed Uninfected Infant
Source: Front Immunol. 2017 Dec 7;8:1704. doi: 10.3389/fimmu.2017.01704 (PMC5725436; doi:10.3389/fimmu.2017.01704)
Supplement: Supplementary file 3 [file Table_1.DOCX]

**Supplementary Table 1. Serology results of the index case.**

|  | **Antenatal serology** (**mother**) **^a^** | | **Baby girl ^b^** | |  |
| --- | --- | --- | --- | --- | --- |
| **Test** | **Values** | **Result** | **Values** | **Result** | **Reference values** |
| Anti-Cytomegalovirus IgG | 171.8 UA/mL | Positive | 49.6 UA/mL | Positive | <6 UA/mL |
| Anti-Rubella IgG | 84.3 UI/mL | Positive | 13.2 UI/mL | Positive | >10 UI/mL |
| Anti-Toxoplasmosis IgG | 163.7 UI/mL | Positive | 19.6 UI/mL | Positive | >3 UI/mL |
| Anti-Herpes I and II IgG | 24.6 | Positive | 6.7 | Positive | >1,25 |
| Anti-Cytomegalovirus IgM | 0.12 | Negative | 0.77 | Negative | <0.85 |
| Anti-Rubella IgM | 0.18 | Negative | 0.06 | Negative | <1.20 |
| Anti-Toxoplasmosis IgM | 0.20 | Negative | 0.29 | Negative | <0.5 |
| Anti-Herpes I and II IgM | Non-reactive | Negative | Non-reactive | Negative | <0.75 |
| VDRL | Weakly reactive | Negative |  | ND^d^ |  |
| Anti-HIV 1 e 2 | 88 nm | Positive |  | Negative | >1 |
|  |  |  |  |  |  |
|  | **Postnatal serology** (**mother**) ^c^ | |  |  |  |
| **Test** | **Values** | **Result** | **Reference values** |  |  |
| Anti-Zika virus IgG | 6 UR/mL | Positive | >1,09 |  |  |
| Anti-Dengue virus IgG | Non-reactive | Negative |  |  |  |
| Anti-Chikungunya virus IgG | <0.8 | Negative | <0.8 |  |  |
| Anti-Zika virus IgM | 0.04 | Negative | <0.8 |  |  |
| Anti-Dengue IgM | Non-reactive | Negative |  |  |  |
| Anti-Chikungunya IgM | Non-reactive | Negative |  |  |  |

^a^ during the 1^st^ trimester

^b^ at two months of age

^c^ six months after delivery

^d^ not performed
